# X-ray Micro-Computed Tomography for Nondestructive Three-Dimensional (3D) X-ray Histology

**DOI:** 10.1016/j.ajpath.2019.05.004

**Published:** 2019-08

**Authors:** Orestis L. Katsamenis, Michael Olding, Jane A. Warner, David S. Chatelet, Mark G. Jones, Giacomo Sgalla, Bennie Smit, Oliver J. Larkin, Ian Haig, Luca Richeldi, Ian Sinclair, Peter M. Lackie, Philipp Schneider

**Affiliations:** ∗ μ-VIS X-ray Imaging Centre, Faculty of Engineering and Physical Sciences, University of Southampton, Southampton, United Kingdom; ‖ Engineering Materials Research Group, Faculty of Engineering and Physical Sciences, University of Southampton, Southampton, United Kingdom; ∗∗ Bioengineering Science Research Group, Faculty of Engineering and Physical Sciences, University of Southampton, Southampton, United Kingdom; † Biomedical Imaging Unit, Faculty of Medicine, University of Southampton, Southampton, United Kingdom; ‡ School of Clinical and Experimental Sciences, Faculty of Medicine, University of Southampton, Southampton, United Kingdom; § National Institute for Health Research Respiratory Biomedical Research Centre, University Hospital Southampton, Southampton, United Kingdom; ¶ Nikon X-Tek Systems Ltd., Tring, United Kingdom

## Abstract

Historically, micro-computed tomography (μCT) has been considered unsuitable for histologic analysis of unstained formalin-fixed, paraffin-embedded soft tissue biopsy specimens because of a lack of image contrast between the tissue and the paraffin. However, we recently demonstrated that μCT can successfully resolve microstructural detail in routinely prepared tissue specimens. Herein, we illustrate how μCT imaging of standard formalin-fixed, paraffin-embedded biopsy specimens can be seamlessly integrated into conventional histology workflows, enabling nondestructive three-dimensional (3D) X-ray histology, the use and benefits of which we showcase for the exemplar of human lung biopsy specimens. This technology advancement was achieved through manufacturing a first-of-kind μCT scanner for X-ray histology and developing optimized imaging protocols, which do not require any additional sample preparation. 3D X-ray histology allows for nondestructive 3D imaging of tissue microstructure, resolving structural connectivity and heterogeneity of complex tissue networks, such as the vascular network or the respiratory tract. We also demonstrate that 3D X-ray histology can yield consistent and reproducible image quality, enabling quantitative assessment of a tissue's 3D microstructures, which is inaccessible to conventional two-dimensional histology. Being nondestructive, the technique does not interfere with histology workflows, permitting subsequent tissue characterization by means of conventional light microscopy–based histology, immunohistochemistry, and immunofluorescence. 3D X-ray histology can be readily applied to a plethora of archival materials, yielding unprecedented opportunities in diagnosis and research of disease.

Living tissues are multiscale three-dimensional (3D) arrangements of cells and tissue matrices that constitute the fundamental building blocks of organs and organ systems. Imaging of such complex tissue architectures on a macroscopic and microscopic level is essential to elucidate their structure-function relationships and to understand the underlying tissue physiology and pathology. At a macroscopic level, where the whole body or large areas, such as whole organs, are of interest, imaging techniques, such as clinical computed tomography (CT), magnetic resonance imaging, and ultrasound imaging, allow for 3D, volumetric imaging. At the tissue and cellular level, imaging is overwhelmingly constrained to two-dimensional (2D) examinations, with light microscopy being the dominant imaging technique for assessment of microscopic tissue structures.

Conventional 2D histology by light microscopy is used to study tissue sections a few micrometers thick, which have been stained histochemically or immunohistochemically through chromogenic or fluorescent labeling. This allows specific tissue and cellular components to be identified and localized and is used to classify a wide range of tissue conditions and disease states. This can inform the stratification of patients for appropriate treatments [eg, for individuals experiencing idiopathic pulmonary fibrosis (IPF)].^1^ Formalin-fixed, paraffin-embedded (FFPE) tissues have been routinely prepared for 2D histopathologic investigation since the end of the 19th Century,^2^ and this approach remains the preponderant tissue preparation method. Although conventional histologic analysis offers high spatial resolution down to a subcellular level, light microscopy of mechanically prepared thin sections can only provide 2D snapshots of the tissue structure. Tissue heterogeneities and structural interconnections are difficult to assess reliably. As a result, for any 3D spatial relationships to be inferred, multiple serial sections need to be cut, prepared, imaged, and then reconstructed,^3^, ^4^ which often requires sophisticated image registration algorithms.^5^

At microstructural or histologic length scales (approximately 1 to 100 μm), there is a lack of 3D analytical platforms that can resolve 3D spatial relationships for both hard and soft tissues. X-ray microcomputed tomography (μCT) or microfocus computed tomography is conceptually equivalent to medical CT, where hardware characteristics and arrangements are optimized for high spatial resolution (in the order of 1 to 100 μm), typically used for imaging material and tissue samples *ex vivo* and *in situ*, with typical sample dimensions in the order of millimeters to centimeters. In keeping with medical CT, μCT imaging is accomplished by placing the sample in the X-ray beam path and capturing projected X-ray absorption patterns (radiographs) over a large number of different rotation angles (typically hundreds to thousands). Contrary to medical CT, where the X-ray source and the detector rotate in a gantry system around the patient, in μCT systems, the X-ray source and detector are usually fixed in place and it is the sample that is rotated during image acquisition. On completion of a scan, CT reconstruction algorithms are used to derive the X-ray absorption of the sample.^6^ The technique was initially developed and optimized to image mineralized bone structures at a microscopic level^7^; and since then, μCT is used routinely in many fields, including archaeology,^8^, ^9^ biomedical research,^10^, ^11^, ^12^, ^13^, ^14^, ^15^, ^16^, ^17^ engineering,^18^, ^19^ materials science,^20^, ^21^ and paleontology.^22^, ^23^ In the biomedical field, μCT has been successfully used over the past two decades to image biological tissues *ex vivo*.^24^, ^25^, ^26^ Soft tissue imaging applications have also been reported, but these mostly rely on laborious and intrusive sample preparation protocols that entail the use of X-ray attenuating stains (eg, osmium tetroxide or iodine),^12^, ^25^, ^27^, ^28^ complex X-ray optics systems, and/or synchrotron light sources.^29^, ^30^

The fundamental challenge for accessible μCT imaging of routinely prepared soft tissues is the inherently low X-ray absorption contrast of these specimens.^12^, ^25^ Mineralized tissues, such as bones and teeth, absorb a large fraction of the incident X-ray photons, resulting in good image contrast, even at typical hard X-ray energy levels offered by laboratory-based μCT systems (peak electrical potentials across the X-ray tube in the range of 20 to 200 peak kV). In contrast, FFPE histology specimens of soft tissues or demineralized hard tissues, with inherently low X-ray attenuation contrast between the tissue and the supporting matrix (paraffin wax), have previously been considered beyond the reach of routine μCT imaging. As noted above, specialized sample preparation protocols and X-ray systems can be used,^12^, ^31^, ^32^ associated with several important disadvantages. For instance, X-ray contrast agents often lack binding-specific affinity for different tissue types and rely on the diffusion of heavy ions (ie, the contrast agent) into the tissue. The latter is a slow process that requires immersion of the tissue into the ions' solution, which can take up to several days to complete.^32^ Also, spatial and temporal anisotropy of stains' penetration can result in artificial contrast gradients between the core and the surface of the tissue and in stain-induced shrinkage of the tissue sample.^31^, ^33^, ^34^ This complicates the interpretation, segmentation, and quantitative analysis of the microscopic tissue features of interest, such as epithelial surfaces, lymphatic vessels, or colonic crypt foci. Moreover, many X-ray contrast agents preclude correlative imaging studies as they are incompatible with histochemical and immunohistochemical staining or with techniques such as laser microdissection for subsequent nucleic acid analysis. Hence, tissue staining with X-ray contrast agents significantly limits practicality, sample availability, and subsequent analysis with conventional histologic methods. Most important, all staining protocols for X-ray imaging are disruptive to established histology workflows, as they are time-consuming and add complication with the need for specialized and additional sample preparation protocols.

Recently, we have demonstrated that FFPE soft tissue samples, routinely prepared for light microscopy–based histology, can be imaged nondestructively using conventional X-ray attenuation–based μCT without the need of any X-ray contrast agents. This was achieved by proposing a μCT imaging protocol at low X-ray fluxes and energy levels that exploits a modest X-ray attenuation contrast window between the soft tissue and the paraffin wax embedding medium.^35^, ^36^ Initially applied to human lung surgical biopsy specimens, the technique had allowed visualization and segmentation of 3D structures^35^ and provided sufficient image contrast for correlative identification and 3D localization of fibroblastic foci in interstitial lung disease (ILD),^36^ evidencing that nondestructive 3D imaging can be used to image soft tissue microstructures in health and disease. Specifically, Jones et al^36^ demonstrated that fibroblastic foci in IPF are independent, discrete structures, in contrast to the previously proposed concept of an extended and interconnected fibroblast reticulum.^37^ These studies highlight the potential of 3D tissue volume analysis by suitably optimized conventional μCT imaging to enhance pathologic understanding and augment the value of correlated 2D imaging results from other histo(patho)logic methods.

In light of these recent steps forward, we developed a bespoke μCT scanner that is optimized for 3D imaging of unstained soft tissues. The new μCT system (Med-X; Nikon X-Tek Systems Ltd, Tring, UK) is designed for use in a medical/clinical environment and combines high-stability X-ray hardware with a high-efficiency detector, providing streamlined high-contrast imaging of routinely prepared soft tissue (ie, standard FFPE blocks) at resolutions in the order of 5 to 10 μm (Figure 1). It is tailored to fit seamlessly into current histology workflows in biomedical and preclinical research, as well as clinical histopathology. We identify this framework as 3D X-ray histology. Herein, we apply 3D X-ray histology to human lung biopsy specimens to demonstrate its promising potential for adding value to the conventional workflow of tissue analysis by (2D) light microscopy.

**Figure 1:**
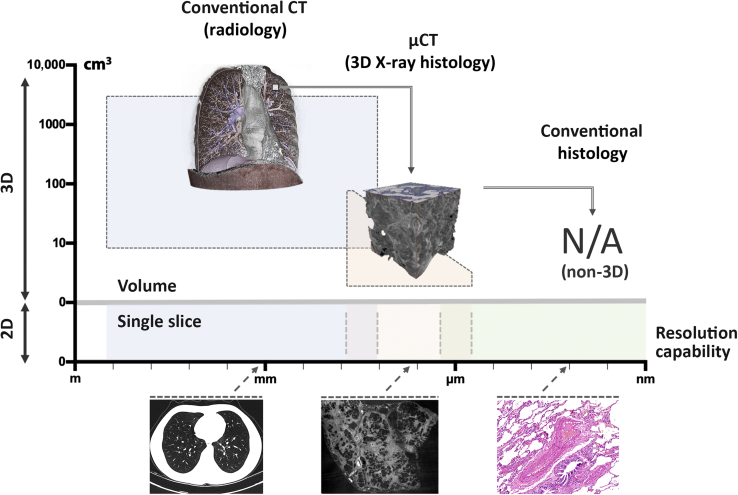
High-resolution medical CT, 3D X-ray histology, using μCT and conventional histology. In lung pathology, anatomic imaging is performed by high-resolution medical CT (**left panel**), whereas cellular analysis is conducted by conventional histology (**right panel**). μCT imaging (**middle panel**) bridges the gap between these traditional imaging modalities, allowing for 3D analysis of biopsy samples at microscopic resolutions. **Left panel** reprinted with permission from Jones et al.^36^**Middle panel** reprinted with permission from Wikimedia commons, Mikael Häggström. NA, not applicable.

## Materials and Methods

### Ethics

The study was performed in accordance with the University of Southampton's (Southampton, UK) ethics policies and ethical guidelines. All samples were obtained with informed consent under full ethical approval (Mid and South Bucks Research Ethics Committee, MREC number 07/H0607/73).

### Human Lung Biopsy Specimens

**Figure 2:**
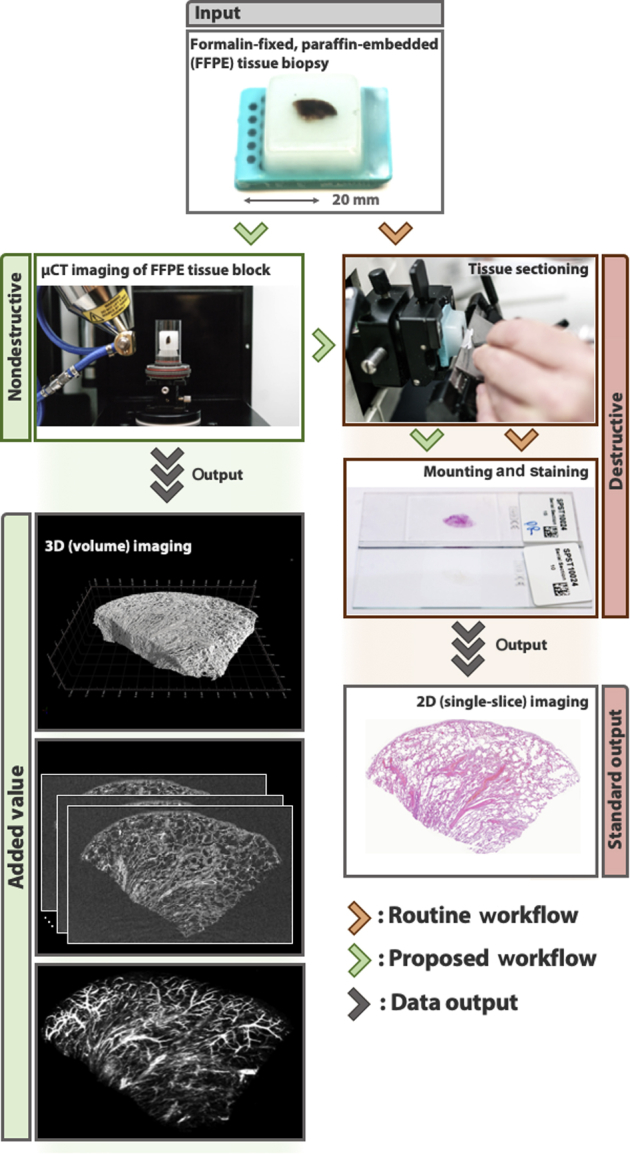
Workflow for 3D μCT imaging and conventional 2D histology. The figure demonstrates the added value of nondestructive 3D imaging to the conventional workflow of tissue analysis by light microscopy, providing high-resolution 3D data that can be integrated seamlessly into protocols for conventional 2D histology.

Two representative human surgical lung biopsy specimens from clinically well-characterized patients with linked records, including clinical diagnosis, were used as the exemplar model for the proposed soft tissue–optimized μCT approach for 3D X-ray histology. A control lung tissue sample was from macroscopically normal lung from a patient undergoing surgery for benign lung nodule resection. A diagnostic surgical lung biopsy sample had a typical usual interstitial pneumonia pattern, confirmed by the independent review of two expert pulmonary pathologists, with the patient subsequently receiving a multidisciplinary diagnosis of IPF. All samples had received routine tissue processing for histology, including formalin fixation (in neutral-buffered formalin) and paraffin embedding (FFPE), and subsequent mounting on standard histology cassettes (Figure 2). Typical lateral dimensions of embedded lung tissue biopsy specimens were in the order of a centimeter, with a thickness in the millimeter range, specifically 11 × 7 × 3 mm for the control and 15 × 8 × 3 mm for the IPF sample.

### μCT Scanner for 3D X-ray Histology

We have developed a novel μCT scanner (Med-X), optimized for soft tissue image contrast. This technology development was a collaborative effort between Nikon X-Tek Systems Ltd and a partnership between the μ-VIS X-ray Imaging Centre at the University of Southampton and the Biomedical Imaging Unit at Southampton General Hospital (Southampton, UK). The project was funded by a Wellcome Trust Pathfinder Award, which was focused on enhanced diagnosis and prognosis in ILD (2016 to 2017). The scanner was built to allow for stable, X-ray absorption–based imaging of unstained FFPE samples with dimensions used in standard clinical cassette mounts. The Med-X system was installed and commissioned in August 2016 at the Southampton General Hospital. The scanner is equipped with a 130-peak kV multimaterial target X-ray source and a high dynamic range 2000 × 2000 pixel flat panel detector.

### μCT Imaging Protocol

To minimize interference of the cassette with the X-ray beam, FFPE lung tissue blocks were decoupled from the histology cassettes by removing excess wax from the back of the cassette and by carefully lifting off the specimen (Figure 2). This ensured that the tissue remained undisturbed and allowed for easy reattachment of the wax block onto the cassette for further processing. The specimens were then placed into thin-walled stackable acrylic polymer cylinders (wall thickness, approximately 1 mm) (Figure 2) and stabilized with polyethylene foam (with negligible X-ray absorption). To increase sample throughput, two cylinders were arranged on top of each other and scanned sequentially in a batch mode.

μCT imaging was conducted using a molybdenum target, the acceleration voltage was set at 55 peak kV, and no X-ray prefiltration was used. The filament current was fixed at 125 μA (resulting in a filament power of 6.9 W); and the source-to-object and source-to-detector distances were set to 42.1 and 992.0 mm, respectively, resulting in an isotropic voxel size of 8.48 μm. The experimental settings were adjusted to maximize the signal/noise ratio and contrast/noise ratio, while ensuring a sample throughput of two specimens per day (9.5 hours of scanning time per sample). A total of 3501 projections were collected over an angular range of 360 degrees, and four frames were averaged per projection to improve the signal/noise ratio. Integration time per projection was set to 2 seconds, and the detector's analogue gain was set to 24 dB.

After μCT acquisition, the data were reconstructed to 32-bit raw volume files by means of Nikon's CT reconstruction software (CTPro version V5.1.6054.18526; Nikon X-Tek Systems Ltd) using conventional filtered back projection.

### μCT Data Processing and Calibration

#### Image Preprocessing

The reconstructed 32-bit raw volumes were imported into Fiji/ImageJ software version 1.51n (NIH, Bethesda, MD; *http://imagej.nih.gov/ij*),^38^, ^39^ where a 3D median filter (1 × 1 × 1 kernel) was applied, followed by a 2D unsharp mask (Gaussian blur factor = 2 pixels, applied on each reconstructed slice of the CT stack). Gray levels were linearly windowed to (−50, 100), containing the X-ray attenuation information of the soft tissue, paraffin wax, and surrounding air, and converted to 16 bit.

#### Image Calibration

The 16-bit CT volumes were calibrated against a custom-made contrast phantom (calibration standard) containing standard histology-grade paraffin wax (Histology Wax; product number 3808605E; Leica Biosystems, Wetzlar, Germany), which was scanned using the same experimental settings before imaging of the actual samples. For this process, the histogram of the central CT slice (relative to the rotation axis) of each volume, containing soft tissue, paraffin wax, and air, was analyzed. The mean gray values corresponding to air and the wax were retrieved in both the actual sample and the phantom, and the two following factors were devised:

(1) $$Contrast\;factor=\frac{\left(I_{wax}-I_{air}\right)}{I_{wax}}$$

(2) $$Calibration\;factor=\frac{Contrast\;factor_{phantom}}{Contrast\;factor_{sample}}$$

where *I*_*wax*_ and *I*_*air*_ in Equation 1 are the mean gray values of the wax and the air, respectively.

The *Contrast factor* expresses the normalized gray value difference (ie, the contrast) between the two known materials in a given scan (namely, air and wax). The *Calibration factor* is a factor the CT data of the sample needs to be multiplied by, so that the contrast in the sample between air and wax matches the respective contrast in the phantom data. After this contrast calibration, the 16-bit gray values of the sample's CT data were linearly offset so that the mean value of air was assigned 0. The resulting calibrated CT volume was then saved as a single 16-bit tiff stack file for visualization, further image processing, and quantification.

### Histology Slide Preparation

After μCT image acquisition, FFPE tissue blocks were reattached to their respective cassettes for routine histologic processing. Sections (4 μm thick) were then cut to a minimum depth of 80 μm into the tissue block and mounted onto glass slides by following standard histology protocols. Finally, sections were deparaffinized and stained using hematoxylin and eosin for later coregistration of the 2D (light microscopy–based) histology slide images with the μCT data.

### Histology Slide Imaging, Digitization, and Coregistration with μCT Data

Histology slides were imaged using a 20× objective on a dotSlide scanning system (VS110 Virtual Microscopy System; Olympus Corp., Southend-on-Sea, UK), visualized using the proprietary VS Desktop software version 2.9 (Olympus Corp.), and saved in the Olympus native .vsi file format. The histology images were then imported in the visualization and analysis software Amira version 6.1.1 (Thermo Fisher Scientific, Waltham, MA), along with the corresponding μCT data sets. Plane correspondence between 2D histology sections and 3D μCT data was achieved by means of elastic landmark-based registration, as follows: i) Using the 2D histology image as a reference, a minimum of three landmark features contained in the histology slide were visually identified in the μCT volume. ii) Because the wax blocks have been mounted parallel to the rotation axis of the μCT scanner (Figure 2), providing reconstructed CT data that are orthogonal to the data from standard histology, the μCT volume was resampled orthogonally to the plane defined by the landmarks using bicubic interpolation. iii) On plane alignment, the CT slice, which matched best the corresponding histology slide, was visually identified, extracted, and imported to Fiji/ImageJ software version 1.51n, along with the histology image. iv) The Fiji plugin *UnwarpJ* (*http://bigwww.epfl.ch/thevenaz/unwarpj*, last accessed June 18, 2019), an elastic registration method based on vector-spline regularization,^40^ was applied to elastically register the histology image (warped source image) to the μCT slice (target image) to account for physical distortions caused during mechanical sectioning of the tissue block.^41^, ^42^

### Visualization of Correlative Histology and μCT Data

Coregistered μCT and histology data visualization was performed in Amira. For this, the calibrated μCT volume was imported into the software, along with the corresponding (coregistered) histology image. A clipping/cropping box was then applied to the μCT data to limit the field of view to 1.2 × 1.2 × 1.2 mm, allowing for a volumetric overview of the tissue microstructure. The μCT volume was windowed in such a way that only the soft tissue components were rendered, excluding air and paraffin wax. In addition, maximum intensity projection (MIP) volume renderings, single slice renderings, and orthogonal slice renderings were performed using The Horos Project version 2.0.2 (Nimble Co. LLC d/b/a/ Purview, Annapolis, MD; *https://horosproject.org*, last accessed June 18, 2019), an open-source medical image viewer based on OsiriX (Pixmeo, Bernex, Switzerland). The 3D MIP tool in Horos has been used to display the 3D volume, with window width and window length altered using the Window Level Option tool to highlight (X-ray) dense structures within the tissue. The same settings have been applied to all images presented herein.

### Quantification of Tissue Microstructural Changes

Microstructural characteristics of the lung tissues were quantified using Fiji/ImageJ software version 1.52d. For this, a mask was generated by manually tracing the boundary of the tissue and restricting the quantification to a volume of interest containing the tissue. Each volume was then binarized by absolute thresholding, using identical threshold gray values for both data sets, to segment (label) the voxels associated with the tissue and to separate them from the voxels in the volume associated with air or wax. The tissue's mean thickness and volume fraction were assessed using the Thickness and the Volume Fraction tools, respectively, of the BoneJ plugin.^43^ By definition, the tools assume that the studied structure (ie, the segmented tissue) is assigned the foreground 8-bit gray value (255) in the binarized image. The thickness of the structure is defined as a volume-weighed arithmetic mean of the local thickness distribution by fitting maximal spheres for all points within the structure, following the method by Hildebrand and Rüegsegger^44^ to assess thickness in 3D, where the local thickness at any given point of the structure is defined as the diameter of the largest sphere that contains this point and fits entirely within the structure. The volume fraction was calculated as the volume of the structure (ie, the segmented tissue), divided by the mask (ie, the volume of interest enveloping the tissue).

## Results

### Correlative Imaging

**Figure 3:**
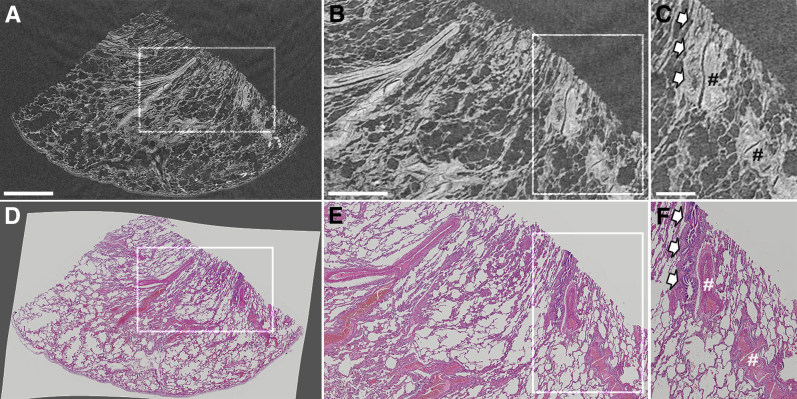
Comparison between lung tissue imaged by μCT and light microscopy for conventional histology. The lung biopsy sample has been taken from a noninvolved site from a patient with lung cancer. **A**–**C:** The μCT slice is provided. **D**–**F:** Digitized images of the histology slide. **D:** The histology image is intentionally presented against a gray background to highlight the degree to which the image had to be unwrapped to fit its nondistorted state (ie, before sectioning). The comparison shows that tissue lung microstructure is clearly visible in the μCT data set at high image contrast levels, which is because of the newly developed μCT scanner that is optimized for soft tissue imaging at high spatial resolutions. **Boxed areas** are shown at higher magnification to the right. **B**, **C**, **E**, and **F:** Higher-magnification images for comparison of μCT imaging to the higher-resolution light microscopy histology. **C** and **F: Arrows** indicate an airway; **hash marks**, a blood vessel, respectively. Scale bars: 2 mm (**A**); 1 mm (**B**); 500 μm (**C**).

Figure 3 shows a representative slice of the reconstructed 3D μCT data set of the FFPE lung biopsy specimen, taken from a noninvolved site from a patient diagnosed with lung cancer, alongside the corresponding 2D hematoxylin and eosin tissue histology slide. This side-by-side presentation of the μCT and histology data (Supplemental Video S1) allows histology-guided identification of a range of tissue structures and diagnostically relevant histologic criteria. In this example, key microstructural features, such as small airways, blood vessels, and alveoli, can be clearly seen in the μCT images and cross-referenced against the histologic sections.

**Figure 4:**
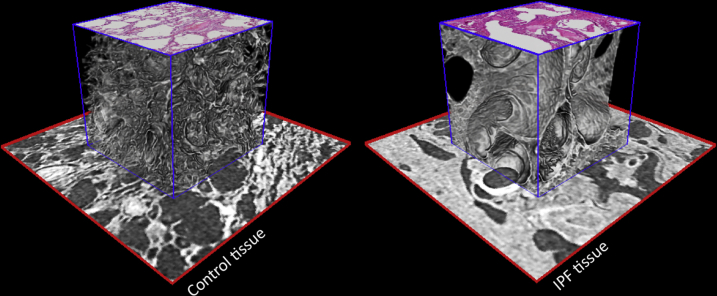
Hybrid visualization of conventional 2D histology slide and 3D X-ray histology image data. Control tissue (**left panel**) and tissue from a patient with interstitial lung disease [idiopathic pulmonary fibrosis (IPF); **right panel**]. Subsections (**red boxed areas**: 2.4 × 2.4 mm) of the tissue imaged by μCT were extracted, and cubic subvolumes (**blue boxed areas**: 1.2 × 1.2 × 1.2 mm) were rendered that protrude above this surface. Coregistered 2D histology sections are placed on top of the rendered μCT cubes at their correct tissue depth, highlighting the capability to combine or fuse conventional 2D histology slides and 3D X-ray histology data. 3D alveolar structure in control lung (**left panel**) can be directly compared with microstructural changes induced by IPF (**right panel**).

Once the 2D histology sections have been coregistered to the volumetric μCT data, both data sets were fused and could be displayed in a hybrid mode in 3D. This is shown in Figure 4, where the hematoxylin and eosin–stained histology sections of the two lung biopsy specimens were digitally interleaved with the corresponding tissue plane of the μCT volume and rendered along with the μCT data. Herein, this rendering mode was used to demonstrate the microstructural differences between the control and the IPF tissue specimen.

### μCT Data Visualization

**Figure 5:**
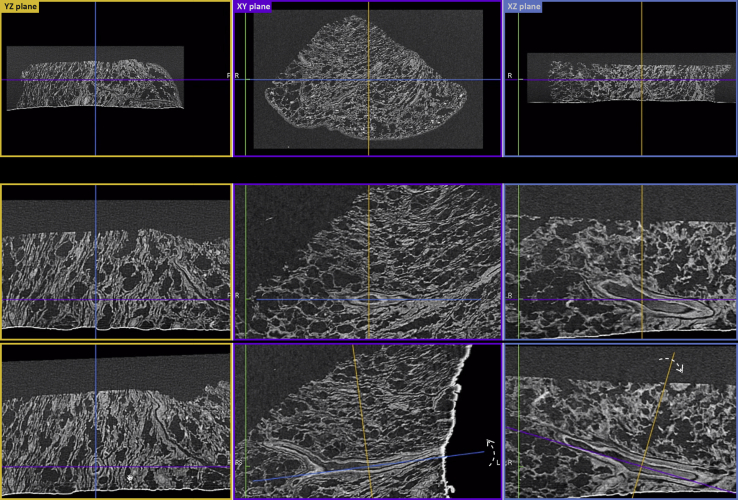
Multiplanar reconstruction of 3D X-ray histology data. **Top row** 3D X-ray histology images can be reviewed immediately after CT reconstruction as an interactive image stack in the three principal orthogonal planes, with the XY plane defined (by convention) as the plane parallel to histology cassette. In this 2D view, the observer can browse through the depth, width, and height of the specimen, zoom, pan, and perform dimensional measurements. The uneven surface of the untrimmed wax can be seen as a bright edge at the bottom of the images shown in the YZ plane and XZ plane. **Middle** and **bottom rows:** Examples of reorientation and reslicing of the specimen to obtain the most relevant histologic (virtual) section. The process can be repeated on-the-fly for multiple features and as many times as required. **Colored lines** indicate the corresponding complementary orthogonal planes (YZ: **yellow lines**; XY: **purple lines**; XZ: **blue lines**); **dashed arrows**, the rotation of the corresponding orthogonal planes with respect to the plane presented in the panel. L, left; R, right.

μCT data can be reviewed immediately after CT reconstruction, presented as an interactive image stack in which the viewer can browse through the depth of the specimen, zoom, pan, and perform basic dimensional measurements (Supplemental Video S2). More importantly, because μCT voxels (3D pixels) are isotropic, orthogonal planes of the μCT data can also be accessed immediately after CT reconstruction. This rendering mode [alias multiplanar reconstruction (MPR)] allows simultaneous imaging of virtual slices taken along the width, depth, and height of the specimen (Figure 5). Because of the isotropic voxel dimensions, virtual reorientation and reslicing of the specimen can be easily achieved *in silico* (Figure 5). An example of such an interactive assessment of the 3D data is shown in Supplemental Video S3, where the reviewer locates an airway and the corresponding blood vessel, examines the cross-sectional views along two orthogonal planes, and proceeds to virtual reorientation and reslicing. By convention, the XY plane is assigned to the plane that is parallel to the histology cassette (Figure 5). In this example (Figure 5 and Supplemental Video S3), rotation of the orthogonal axis viewing system along the *z* and *x* axes was performed to fully expose the longitudinal and transverse cross-sections of the blood vessel in the YZ/XZ and XY planes, respectively.

**Figure 6:**
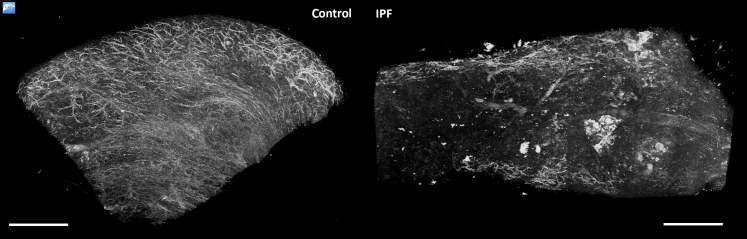
Maximal intensity projections of 3D X-ray histology data. Control tissue (**left side**) and tissue from a patient with interstitial lung disease [idiopathic pulmonary fibrosis (IPF); **right side**]. Snapshots of 3D volumes are shown as maximum intensity projections (MIPs). MIP rendering consists of projecting the pixels with the highest X-ray attenuation, which helps to discern denser features, such as the vascular network, from the surrounding tissue, without the need of data segmentation. MIPs illustrate a clear difference between the vascular network in control tissue (**left side**) and ablated vascular structures observed for the IPF lung tissue (**right side**). Samples were of equivalent size, and identical threshold parameters were applied for both samples. Scale bars = 2 mm.

An alternative and computationally inexpensive method to render volume data are MIPs. MIP rendering is generated by an algorithm that casts rays of light through the data set, where only the voxel with the highest X-ray attenuation (ie, the brightest voxel) along each ray path is rendered.^45^ Figure 6 and Supplemental Videos S4 and S5 illustrate the ability to selectively discern features within a lung biopsy sample using the aforementioned MIP rendering method, applied on the μCT data sets.

### Image Quantification

**Figure 7:**
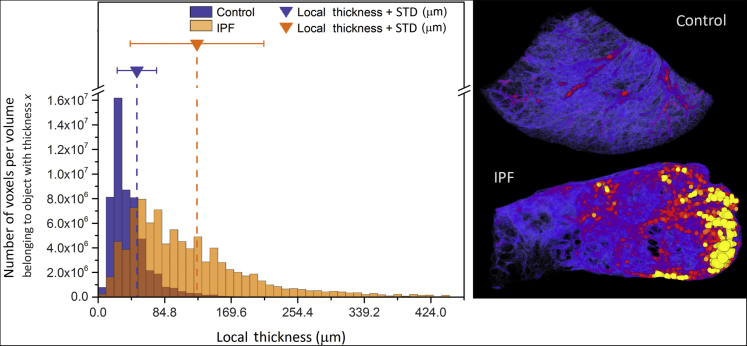
Quantitative analysis of the tissue thickness. Analysis of the local tissue thickness showing the differences between the volumetric characteristics of control and the idiopathic pulmonary fibrosis (IPF) tissue biopsy specimens. On average, control lung tissue was composed of finer elements, with an average thickness of 42 μm (approximately 5-pixel sphere diameter at a voxel size of 8.48 μm), compared with much thicker elements for the IPF tissue biopsy specimen, with an average thickness of 127 μm (approximately 15 pixels). **Left panel:** The histogram depicts the frequency and distribution of all tissue voxels (3D pixels) on the basis of the thickness of the element they belong to. The graph also presents the calculated average (mean) thickness for each specimen (**dashed vertical lines**), along with the associated SD (**solid line whiskers**). **Right panel:** A volume rendering of the thickness map for both specimens. Both color maps are to scale and range from 0 μm (black/blue) to 300 μm (yellow). This view provides a complementary qualitative visualization of the thickness heterogeneity.

Analysis of the tissue thickness showed that differences between the characteristics of the control and the IPF tissue biopsy specimens could be identified. On average, the control lung tissue biopsy specimen contained thinner structures (49.2 μm) than the IPF tissue biopsy specimen (125.6 μm) (Figure 7). Volume rendering of the thickness map also shows greater heterogeneity in the IPF specimen, compared with the control (Figure 7). In terms of volume fraction, the control sample had a smaller volume of lung tissue per unit volume of sample (33%) than the IPF sample (60%).

### Stability of the μCT Scanner

**Supplemental Figure S1:**
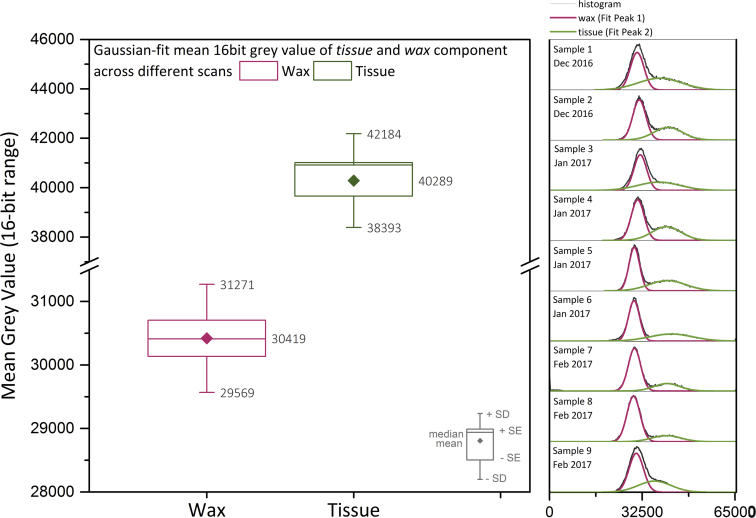
Stability of the new μCT scanner for 3D X-ray histology. **Left panel:** Gray value variation for both the paraffin wax (purple) and lung tissue material (green) of 30 scans (not shown in this study) after calibration, spanning a 3-month μCT imaging period. **Right panel:** Raw 16-bit histograms (**black lines**) of central slices of nine representative specimens spanning the duration of the aforementioned period, together with the corresponding fitted (Gaussian) peaks for the wax (purple) and tissue material (green) after calibration.

The μCT imaging and calibration protocol has been applied for >30 lung biopsy samples (not shown here), resulting in consistent and reproducible image quality characteristics. Supplemental Figure S1 shows the histograms of nine specimens spanning a 3-month imaging period, along with the gray value variation for both the paraffin wax and tissue components of these specimens after gray value calibration. The graph demonstrates that fluctuation of the mean gray value for wax (30,419) was always <1% (SEM = 284). The fluctuation of the mean gray value (40,289) for the tissue was higher but <2% (SEM = 632), which is to be expected, given the electron density inhomogeneity of biological material.

### Data Availability

All data supporting this study are openly available from the University of Southampton repository (*https://doi.org/10.5258/SOTON/D0902*).

## Discussion

The study presented herein is targeted at delivering a 3D visualization solution for X-ray histology that is compatible with current histology workflows. It summarizes the results of >3 years of continuous development of hardware technology as well as imaging and visualization protocols and workflows since our first proof-of-principle μCT study on 3D imaging of paraffin-embedded human tissue samples.^35^ The prototype μCT system we developed and used herein (Med-X) represents a distilled version of its engineering ancestor (a heavy-duty walk-in 4.6 × 2.4 × 3.3-m experimental chamber used in our previous work^35^) and was specifically designed and optimized for use in a medical/clinical context and environment. Our study presents a detailed technical setup for 3D X-ray histology and establishes a detailed workflow specifically aligned with the use of paraffin-embedded tissue samples, which are routinely prepared for research or diagnostic applications. This includes assessment of equipment performance and stability over a period of several months; and it critically includes a calibration procedure that establishes a baseline, which can serve as a standard for μCT imaging of soft tissues. This allows direct cross-sample and cross-scan comparisons and, in the future, also cross-site comparisons between different laboratories operating similar μCT systems. Briefly, calibrated 3D X-ray histology allows comparable 3D soft tissue μCT data sets to be generated over time and in different laboratories and opens up tissue X-ray densities and microstructures to be scrutinized in a quantitative manner.

X-ray attenuation–based μCT imaging of soft tissues without the use of any X-ray contrast agents is made possible through 3D X-ray histology. Direct comparison of the *de facto* standard for microstructural soft tissue analysis (namely, 2D light microscopy–based histology) with 3D X-ray histology will be pivotal for the validation and adoption of the technique by histologists and histology laboratories. This approach is likely to be of particular value in conditions such as ILD, where diagnosis is often challenging and pathologic changes are dispersed and heterogeneous. The ability to identify the 3D location and extent of interstitial fibrosis and, in particular, fibroblastic foci within IPF samples using this approach has been demonstrated by Jones et al.^36^ Being able to review larger tissue volumes than is easily possible by conventional histology should facilitate the identification and assessment of spatial heterogeneity, patchy involvement of lung parenchyma, and the presence of architectural distortion or microscopic honeycombing in ILD.^46^ Using this nondestructive technique with standard FFPE samples opens up the possibility of applying soft tissue–optimized μCT in a clinically relevant context (Figure 1).

### 3D X-ray Histology

#### Visualization Modes and Applications

Building on μCT studies of paraffin-embedded human tissue samples we published previously,^35^, ^36^ we discuss and demonstrate herein a range of visualization options for 3D X-ray histology using established medical image viewers. Our aim was to demonstrate the potential to visualize the information-rich 3D data of (human) tissue, displaying microstructures in more detail and in a way that is relevant to biomedical research and medical/clinical applications.

#### Distortion-Free Imaging and Correlative 2D and 3D Visualization

Sectioning-induced defects, also referred to as cutting, preparation, or histologic artifacts, are a common problem encountered with physical cutting of FFPE tissue blocks in histology (ie, microtomy), which can distort the tissue structure.^47^ 3D X-ray histology data can be used as a reference point to review the tissue in its initial state, before physical sectioning, which induces tissue distortion and defects.^48^ Correlative 3D μCT-2D histology renderings can be used to assess the extent and location of tissue loss, damage, and distortion induced during histology slide production.^48^, ^49^, ^50^ More importantly, these hybrid image rendering modes provide novel and unique ways to cross-reference and analyze microstructural tissue details in 2D and interpret them in a 3D context. Coregistered histology and volumetric μCT data put in context the conventional histology images within the 3D structure that is depicted by the μCT data. However, at this stage of multimodal image fusion, coregistration of the volumetric μCT data and 2D histology required a moderate amount of data handling and manipulation (1 to 2 hours per data set for this study) in specialized 3D volume manipulating software suites.

#### Multiplanar Reconstruction

MPR, on the other hand, is a rendering mode immediately available to the user after CT reconstruction and represents a powerful 3D visualization tool for 3D X-ray histology data. The XY plane image shown in Figure 5 is a virtual section, analogous to what a physical section would have generated. Consequently, scrolling through the XY image stack of virtual sections is equivalent to physical serial sectioning the tissue block to exhaustion, with a slice spacing equal to the CT voxel size (herein, 8 μm). Interactive image stack and orthogonal plane renderings (Supplemental Videos S2 and S3) of the μCT data can be used to provide a detailed overview of tissue microstructure, the histology equivalent of which would require sectioning of the tissue block to exhaustion at huge manpower costs. For instance, a 15 × 15 mm × 1-mm piece of tissue, sectioned at a typical interval of 4 μm, would result in 250 histologic sections if processed throughout its entire depth, whereas the same specimen can be scanned using μCT in just a few hours in 3D and at a voxel size of 8 μm, resulting in >100 virtual sections (8 μm thick).

As shown in Figure 5 and demonstrated in Supplemental Video S3, MPR also enables the user to dynamically adjust the slicing orientation. In a medical context, this can be used for obtaining the most relevant histologic (virtual) section and for examining tissue sections along all three orthogonal planes simultaneously. The resulting data set can be used for analyzing the spatial arrangements of tissue (micro)structures, their orientation, as well as heterogeneity and connectivity in 3D.

From the sample preparation point of view, MPR can be exploited to preview the tissue, define the optimal physical sectioning orientation of the tissue, and guide histologic sectioning for conventional histology (ie, image-guided histologic sectioning).^51^, ^52^ μCT can provide information about the appropriate tissue orientation and presence or absence and location (eg, depth) within the tissue of specific features of interest, a prospect particularly valuable in cases of small, oddly shaped biopsy specimens and/or applications where there is a real risk of missing the relevant tissue depth for histology (eg, tumor margin assessment).

#### Maximum Intensity Projections

The aforementioned MPR rendering mode adds depth information to a 2D image, by simultaneously providing the user with 2D virtual sections along the depth and width of a specific tissue feature. 3D perception is reliant on the user's ability/training. On the contrary, MIP is a 2D representation of the 3D structure. MIP selectively renders the brightest voxels, characteristic for structures with higher X-ray absorption, along a specific path, providing an overview of the spatial density of structures, such as vessels, airways, calcifications, or exogenous deposits. As 3D renderings with MIP are computationally inexpensive with little to no manual input, they can provide an immediate overview of dense structures, such as vessels, airways, or calcifications, for fast volume screening. For instance, the extent of change in the lung vascular network due to disease or anatomy can be visualized and assessed immediately, as demonstrated in Figure 6. MIP rendering is widely used in radiology,^53^ mainly for angiography. However, MIP presentations lack true 3D depth and the viewer cannot discriminate if a certain feature is in front of or behind another one, along the rendered path. This limitation can be mitigated by composing sequential MIPs of the volume at different angular positions by rotating the volume about a predefined axis (Supplemental Videos S4 and S5). When motion is added, for instance by dynamic spatial manipulation or video rendering, the viewer perceives depth information, leading to spatial localization of the rendered features.

### Calibrated Data for the Widespread Application and Adoption of 3D X-ray Histology

For widespread application and adoption of 3D X-ray histology, the technique must also offer calibration protocols for providing reproducible μCT image quality that guarantee comparable results over time and between different laboratories. In clinical CT, this is achieved by regular quality control tests of the CT equipment, which ensure that the achieved image quality fall within an accepted tolerance, depending on the application,^54^ both in terms of random uncertainty in voxel value (noise) and CT number (calibrated voxel gray values; see below).

In this study, we devised a calibration protocol similar to that used for calibrating clinical CT equipment, which sets the CT numbers of water at 0 and air at −1000 in the Hounsfield scale,^6^ but instead of water paraffin wax was used as the reference material. Hounsfield units are used for biological systems, where water constitutes the main component. The selected range of the Hounsfield scale provides a quantitative tool for assessing whether the X-ray attenuation of a given voxel in a CT volume is equal, greater (eg, bones), or lower (eg, fat) than water. However, FFPE specimens of soft tissues are composed of dehydrated tissue, wax, and air, all of which exhibit lower X-ray attenuation than water. As a result, the CT numbers in a μCT image of an unstained FFPE specimen would be negative (Hounsfield units < 0). By selecting the paraffin wax as reference material and by setting air to 0 (as opposed to water) ensured in the current study that the resulting gray values of the calibrated 3D X-ray histology data sets were always positive. Quantitative industrial CT often adopts a similar approach, where the Hounsfield scale is offset by 1000 units and redefined such that air is 0 and water is 1000 (sometimes referred to as industrial Hounsfield units or offset Hounsfield units) (X-TEK: CT Pro 3D User Manual; Nikon Metrology, Hertfordshire, UK).^55^

### Added Value of 3D Imaging in Histology

3D imaging of tissue structures by μCT provides information about spatial heterogeneity (Figures 6 and 7) and connectivity of the tissue,^36^ which is not accessible in 2D. The nondestructive, high-resolution capability of 3D X-ray histology also allows for whole-cassette visualization down to microscopic levels, minimizing the risk of inadequate tissue sampling. Currently, scanning volumes for a voxel size range of 5 to 10 μm vary from approximately 100 to 800 mm^3^ using the imaging protocol presented herein; however, this can be significantly increased with the implementation of alternative acquisition techniques, such as helical cone- or fan-beam μCT.^56^

Disease classification for patient stratification relies on histopathologists with many years of training and experience. Despite this, diseases such as ILD, with extensive tissue heterogeneity and histologic variability, still have poor rates of interobserver agreement, even between experts.^57^ Quantitative microstructural analysis at histologic resolution in 3D is likely to deliver significant added value to the interpretation of pathologic changes. In addition, areas where such pathologic changes are present can be located and related to their 3D context within the tissue volume. 3D X-ray histology can provide increased and novel contextual information in a multiplanar and multiscale format. Structures with different orientations within the tissue architecture can now be viewed as 3D objects *in silico*. Pathologic features and changes in tissue microstructure can be interpreted and analyzed in 3D, as opposed to being limited to 2D sections, uncovering the true extent of tissue dysmorphia.

The results of the analysis presented herein demonstrate that microstructural characteristics, such as tissue thickness and volume fraction, can be assessed by quantitative morphometric measures in 3D, also allowing local heterogeneity to be identified (Figure 7 and Supplemental Videos S6 and S7). At this point, the exemplars of local thickness and volume fraction presented in Figure 7 were used solely to demonstrate the quantitative imaging capability of the technique. Although it is reasonable to assume that both these measures could differ between the control and the IPF tissue, this study was not designed to provide evidence of that. The measured difference might well be due to other factors, such as difference in vascularity and presence of airways.

Nonetheless, the ability to quantify 3D features adds a new family of classifiable measures, which, when paired with artificial intelligence and computer-aided diagnosis systems, could potentially revolutionize diagnostics.^58^ More importantly, being a nondestructive and noncontact technique, 3D X-ray histology is fully compatible with subsequent sectioning for conventional histology.

## Conclusions and Outlook

Current laboratory-based μCT imaging protocols for soft tissue imaging mostly rely on contrast agents and intrusive staining procedures of the tissue, which can adversely affect tissue characteristics, such as immunoreactivity. This represents a significant barrier for the uptake of μCT-based 3D imaging into routine histology workflows. The ability to integrate 3D X-ray histology data sets of standard FFPE samples (Figures 1 and 2) into manageable and timely analysis platforms for more rapid and systematic analysis (Figures 3, 4, 5, and 6) will allow for μCT to become a routine partner in the analysis of soft tissue, both in biomedical research as well as in a medical/clinical context.

In this study, we demonstrated how the presented soft tissue–optimized μCT workflow for 3D X-ray histology can be validated by standard 2D histologic techniques, opening the way for widespread application and adoption in basic research and clinical pathology. As a research tool, nondestructive 3D X-ray histology can be combined with an array of existing 2D histologic techniques, including immunocytochemistry, immunofluorescence, and *in situ* hybridization, leading to better understanding of disease initiation and progression in 3D. In pathology, 3D X-ray histology could help identify new microstructural hallmarks of disease. Notably, it will enable high-resolution 3D imaging to be applied to the plethora of archival FFPE material stored in many hospitals and tissue banks, which will deepen our understanding of disease progression. If combined with patient records, it will allow validation of microstructural hallmarks of disease in terms of their diagnostic and predictive power for soft tissue–related diseases using clinical end points. In a clinical environment, 3D X-ray histology, coupled with artificial intelligence/computer-aided diagnosis, could improve diagnostic accuracy and support patient stratification.

At this stage, further work needs to be performed to define the context in which microscopic 3D volumetric analysis is useful in a medical/clinical environment. We strongly believe that similar to how medical CT and magnetic resonance imaging revolutionized clinical practice in recent decades, 3D X-ray histology will play a pivotal role in research and clinical histology in the near future.

## Supplemental Data

Supplemental Video S1 Simultaneous visualization of a single μCT and hematoxylin and eosin histology slice. Coregistration of histology with μCT image data and simultaneous visualization of both data sets allow for direct comparison of the two imaging modalities and precise, histology-guided identification of a wide range of tissue structures and diagnostically relevant histologic criteria. Visualization platform: Horos version 2.0.2.

Supplemental Video S2 Scrolling through the XY slice-stack (ie, virtual sectioning). μCT data can be reviewed immediately after CT reconstruction. In its simplest case, volume visualization is accomplished within seconds after μCT scan completion on a slice-by-slice basis, presented as an interactive image stack in which the viewer can browse through the depth of the specimen, zoom, pan, and conduct dimensional measurements. Visualization platform: Horos version 2.0.2.

Supplemental Video S3 Orthogonal plane view and arbitrary virtual slicing. Orthogonal planes of the μCT data can also be viewed immediately after CT reconstruction for analyzing the spatial arrangements of tissue (micro)structures, their orientation, as well as heterogeneity and connectivity in (3D) space. An example of such an interactive assessment of 3D data is shown herein, where the reviewer locates a blood vessel and examines the cross-sectional views along two orthogonal planes. Visualization platform: Horos version 2.0.2.

Supplemental Video S4 Maximum intensity projection (MIP) rendering of the control lung tissue biopsy specimen. This 2D representation of a 3D structure provides a good definition of denser structures, such as vessels, airways, calcifications, or exogenous deposits. MIP rendering helps to identify and emphasize particular tissue structures of interest, such as the vascular network. Dynamic MIP rendering for different angular positions (eg, rotating video) conveys qualitative depth information to the observer. Visualization platform: Horos version 2.0.2.

Supplemental Video S5 Maximum intensity projection (MIP) rendering of a lung biopsy sample obtained from a patient with idiopathic pulmonary fibrosis. This 2D representation of a 3D structure shows good definition of denser structures, such as vessels, airways, calcifications, or exogenous deposits. MIP rendering helps to identify and emphasize particular tissue structures of interest, such as the vascular network. Dynamic MIP rendering for different angular positions (eg, rotating video) conveys qualitative depth information to the human observer. Visualization platform: Horos version 2.0.2.

Supplemental Video S6 3D rendering of local thickness of the control lung tissue biopsy specimen. The video presents an overview of the 3D distribution of the local thickness of the control lung tissue biopsy specimen. Each point is colored according to the size of the diameter of the largest sphere that fits inside the structure at this point. Smaller elements of the structure are represented with cooler colors, and bigger ones are represented with warmer colors. The color scale is kept consistent between this video and Supplemental Video S7.

Supplemental Video S7 3D rendering of local thickness of the idiopathic pulmonary fibrosis (IPF) lung tissue biopsy specimen. The video presents an overview of the 3D distribution of the local thickness of the IPF lung tissue biopsy specimen. Each point is colored according to the size of the diameter of the largest sphere that fits inside the structure at this point. Smaller elements of the structure are represented with cooler colors, and bigger ones are represented with warmer colors. The color scale is kept consistent between this video and Supplemental Video S6.
